# Supplementary figures and images for: Bacterial exposure leads to variable mortality but not a measurable increase in surface antimicrobials across ant species
Source: PeerJ. 2020 Dec 3;8:e10412. doi: 10.7717/peerj.10412 (PMC7719289; doi:10.7717/peerj.10412)

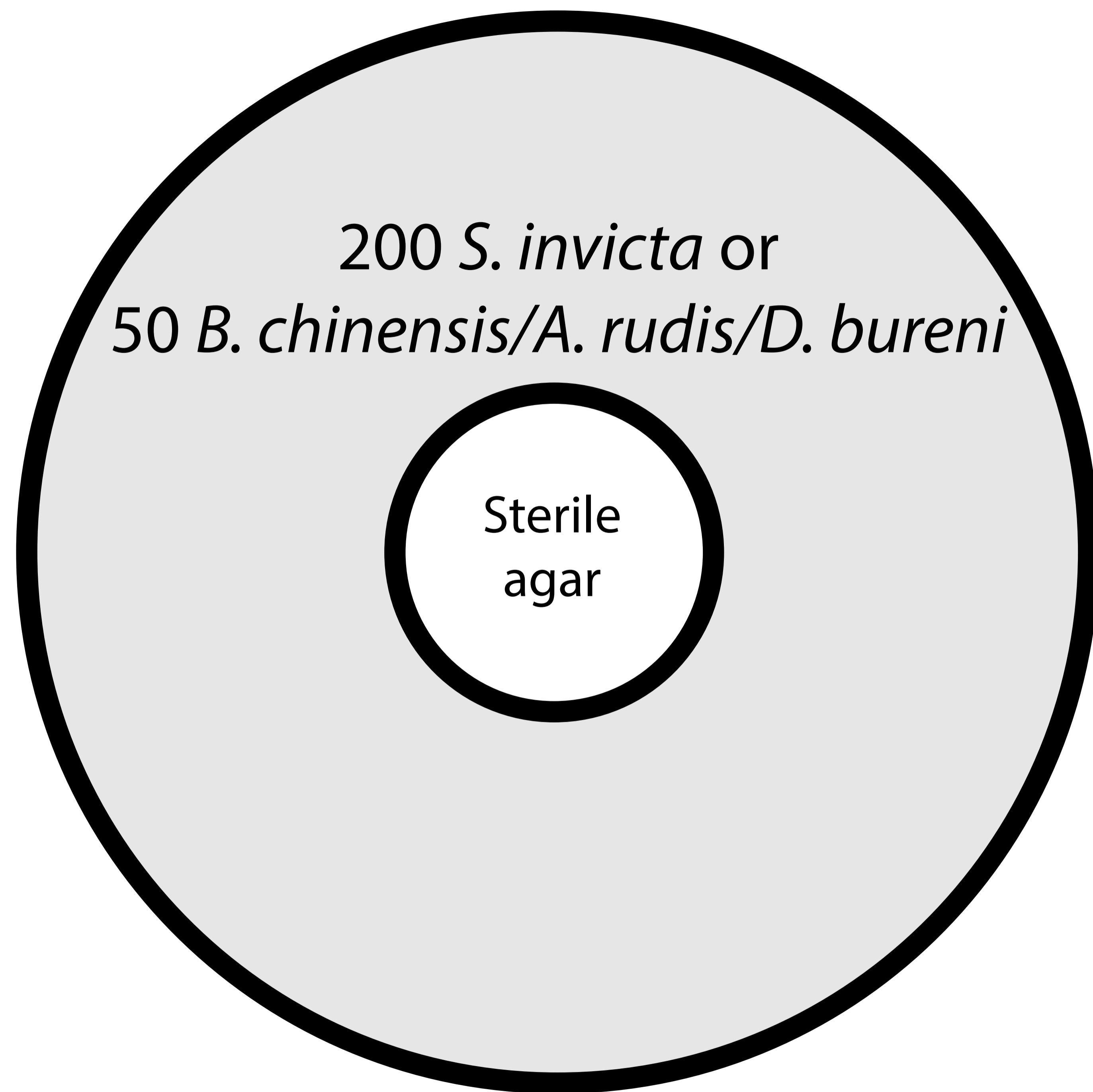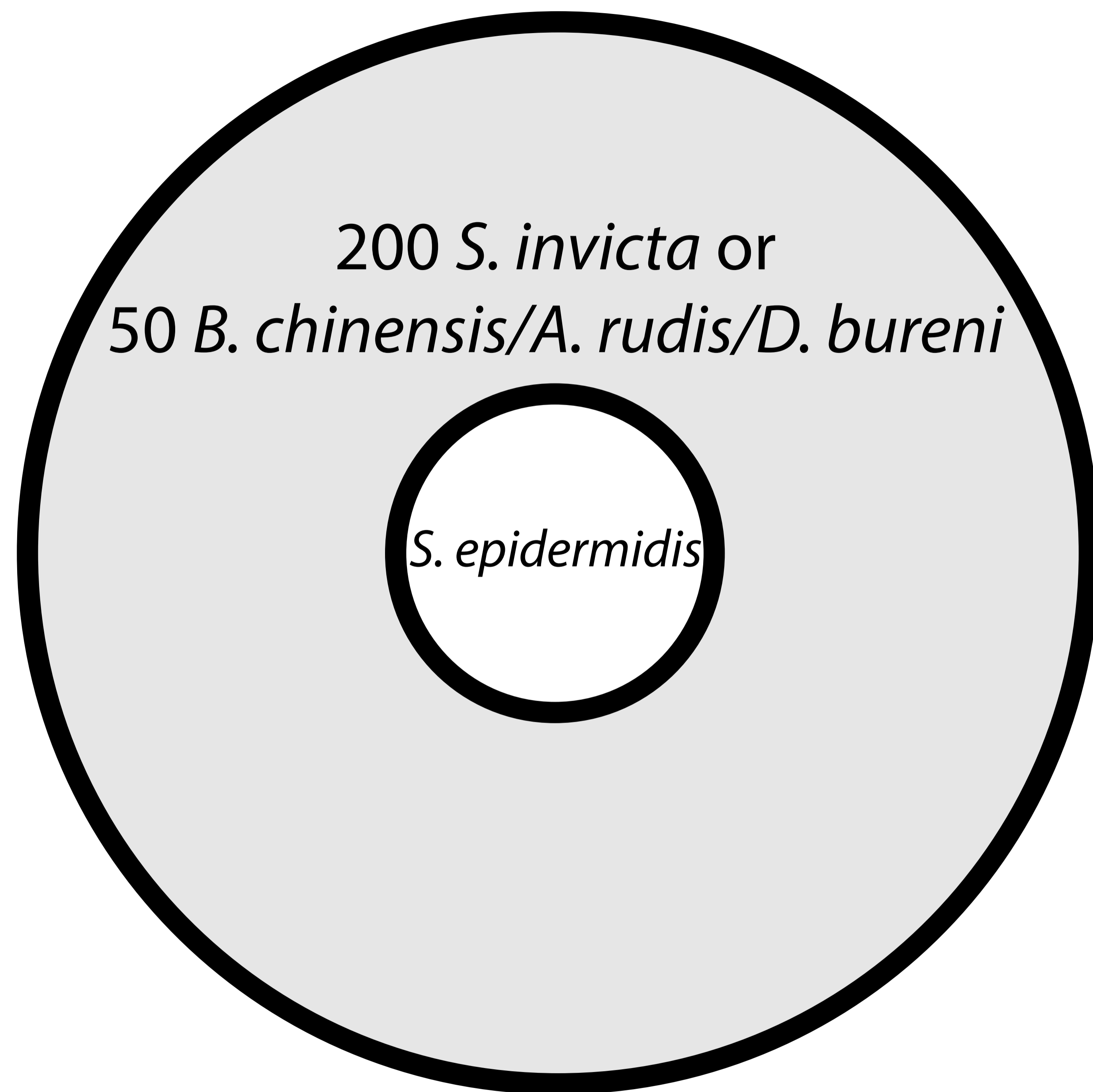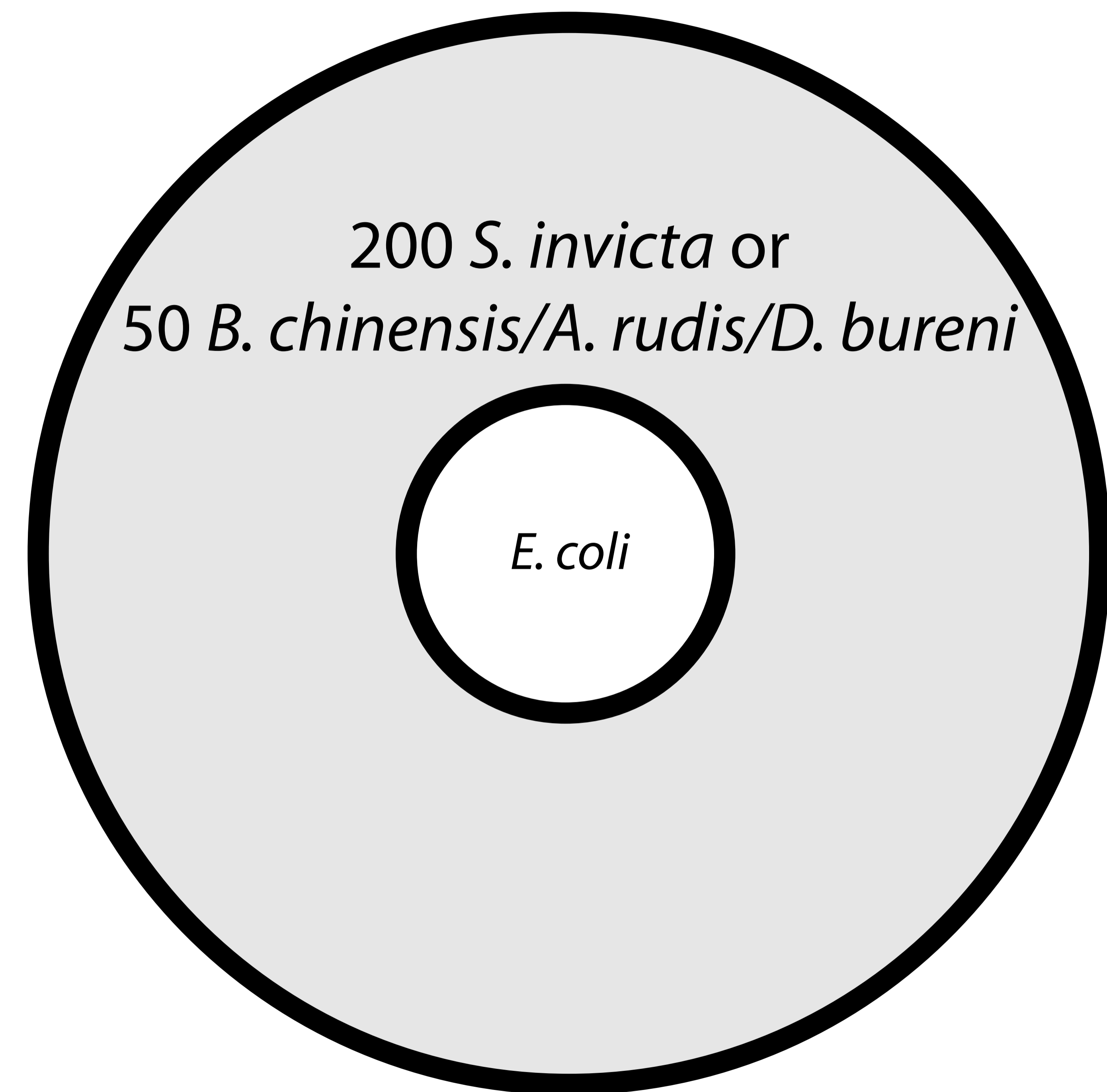

Supplement: Supplemental Information 2 — 15 colonies were tested for each species across all three treatments. Treatments were 48 hr exposure to E. coli or S. epidermidis (0.5 MacFarland standard) or sterile agar with mortality counted at 24 hr and continuously at 48 hr. 200 S. invicta worker ants and 50 workers from B. chinensis, A. rudis, and D. bureni were used in each treatment. 100 ml of water was added under each piece of agar. Figure to scale. [file peerj-08-10412-s002.pdf]
