# Supplementary material for: Bacterial exposure leads to variable mortality but not a measurable increase in surface antimicrobials across ant species
Source: PeerJ. 2020 Dec 3;8:e10412. doi: 10.7717/peerj.10412 (PMC7719289; doi:10.7717/peerj.10412)
Supplement: Supplemental Information 3 — 15 colonies were tested for S. invicta and B. chinensis across all three treatments. Initially, three exposure treatments were set up akin to experiment 1, where 200 workers of S. invicta and 50 workers of B. chinensis were exposed to E. coli or S. epidermidis (0.5 MacFarland standard) or sterile agar, for a 6 hr exposure. Next, 40 living workers from each treatment were collected for surface extraction, where extract was resuspended, filter sterilized, then aliquoted into 10 worker ant equivalent samples. Samples were tested for their antimicrobial ability against E. coli or S. epidermidis in a modified 96 well plate assay that had been used in previous studies to assess antimicrobial ability of insect surface extracts (Stow et al., 2007). This experiment had an unbalanced design as replicates of B. chinensis to be extracted across all treatments did not come from the same colony; that is, each treatment and respective control for a replicate came from two different colonies. [file peerj-08-10412-s003.pdf]

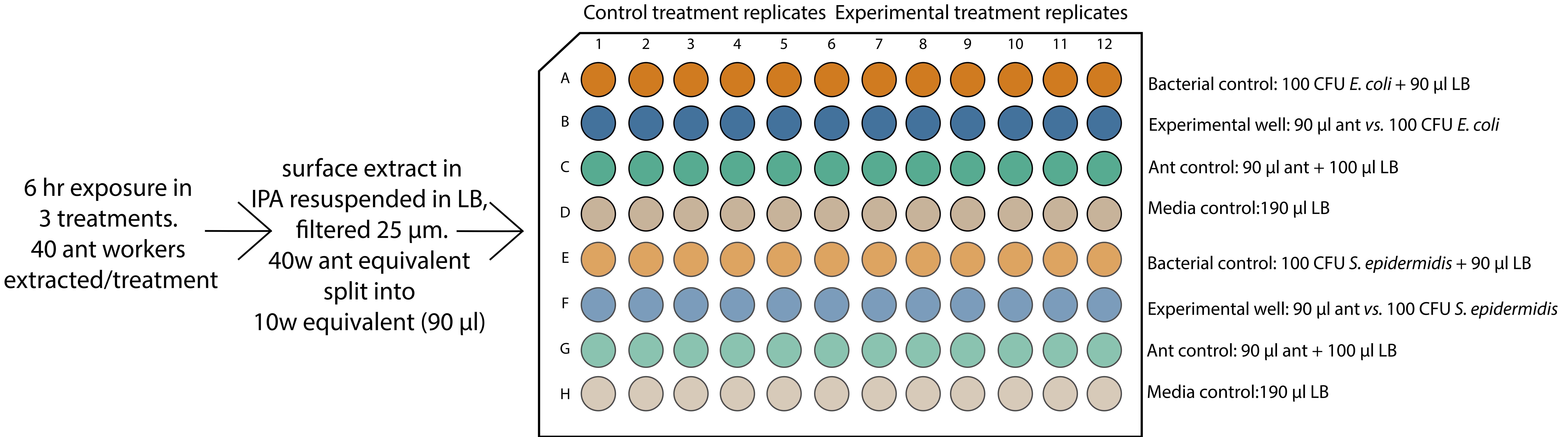

6 hr exposure in
3 treatments.
40 ant workers
extracted/treatment
surface extract in
IPA resuspended in LB,
filtered 25 µm.
40w ant equivalent
split into
10w equivalent (90 µl)
Control treatment replicates
Experimental treatment replicates
1 2 3 4 5 6 7 8 9 10 11 12
A
B
C
D
E
F
G
H
Bacterial control: 100 CFU E. coli + 90 µl LB
Experimental well: 90 µl ant vs. 100 CFU E. coli
Ant control: 90 µl ant + 100 µl LB
Media control:190 µl LB
Bacterial control: 100 CFU S. epidermidis + 90 µl LB
Experimental well: 90 µl ant vs. 100 CFU S. epidermidis
Ant control: 90 µl ant + 100 µl LB
Media control:190 µl LB
